# Beyond Needles: Immunomodulatory Hydrogel-Guided Vaccine Delivery Systems

**DOI:** 10.3390/gels11010007

**Published:** 2024-12-26

**Authors:** Md Mohosin Rana, Cigdem Demirkaya, Hector De la Hoz Siegler

**Affiliations:** 1 Department of Pathology and Laboratory Medicine, Faculty of Medicine, University of British Columbia, Vancouver, BC V6T 1Z3, Canada; mohosin.rana@ubc.ca; 2 Centre for Blood Research (CBR), Faculty of Medicine, University of British Columbia, Vancouver, BC V6T 1Z3, Canada; 3 Department of Chemical and Petroleum Engineering, Schulich School of Engineering, University of Calgary, Calgary, AB T2N 1N4, Canada; cigdem.demirkaya@ucalgary.ca

**Keywords:** vaccine delivery, hydrogels, immunomodulation, adjuvants, immunogenicity, biomaterials

## Abstract

Vaccines are critical for combating infectious diseases, saving millions of lives worldwide each year. Effective immunization requires precise vaccine delivery to ensure proper antigen transport and robust immune activation. Traditional vaccine delivery systems, however, face significant challenges, including low immunogenicity and undesirable inflammatory reactions, limiting their efficiency. Encapsulating or binding vaccines within biomaterials has emerged as a promising strategy to overcome these limitations. Among biomaterials, hydrogels have gained considerable attention for their biocompatibility, ability to interact with biological systems, and potential to modulate immune responses. Hydrogels offer a materials science-driven approach for targeted vaccine delivery, addressing the shortcomings of conventional methods while enhancing vaccine efficacy. This review examines the potential of hydrogel-based systems to improve immunogenicity and explores their dual role as immunomodulatory adjuvants. Innovative delivery methods, such as microneedles, patches, and inhalable systems, are discussed as minimally invasive alternatives to traditional administration routes. Additionally, this review addresses critical challenges, including safety, scalability, and regulatory considerations, offering insights into hydrogel-guided strategies for eliciting targeted immune responses and advancing global immunization efforts.

## 1. Background

Vaccines represent one of the most significant advancements in biomedical science, reducing the prevalence, morbidity, and mortality of infectious diseases like influenza, measles, polio, and smallpox [1]. Beyond individual protection, vaccination campaigns create herd immunity, offering community-wide defense against the spread of infectious agents. Despite these successes, many infectious diseases, including malaria, tuberculosis, and human immunodeficiency virus (HIV), lack effective vaccines [2]. A significant hurdle in developing vaccines is the need for adjuvants able to promote and sustain a targeted immune response. To address these challenges, vaccines increasingly incorporate immunomodulatory adjuvants, biologics that amplify the immune response to antigens [3]. These adjuvants interact with the immune system to provoke a stronger, longer-lasting immune response. Through innovative approaches, vaccines have evolved to include DNA, recombinant subunits, synthetic peptides, and nanoparticles [4,5,6,7]. Each of these additions is intended to stimulate an antigen-specific immune response, aiding the body’s ability to recognize and respond to a pathogen more effectively upon exposure. However, to optimize vaccine efficacy, a reliable delivery system that can target the site of action and promote gradual antigen release has become essential.

Several vaccine delivery systems have been developed to address these needs, including polymer-based systems, nanoparticle-based carriers, and three-dimensional (3D) scaffold-based structures [8,9,10,11]. Each offers unique advantages in delivering antigens to specific anatomical sites and enhancing the interactions with immune cells: polymeric systems encapsulate the antigen and allow for its gradual release [12], maintaining antigen exposure to immune cells over an extended period; nanoparticles can target specific cells or tissues due to their small size and functionalized surface properties [12]; and 3D scaffolds can mimic natural tissues and provide controlled release of antigens [10,13]. However, there are remaining challenges in achieving precise targeting, ensuring safety, and handling diverse antigen chemistries [14]. Addressing these limitations is crucial for advancing vaccines, especially for diseases that require robust, long-term immune responses.

Hydrogels have emerged as a transformative solution for vaccine delivery systems [15]. These polymer-based materials enable precise, localized delivery of antigens while supporting gradual release, prolonging immune activation, and reducing the need for booster doses [16,17,18]. Furthermore, hydrogels inherently act as adjuvants, amplifying the immune response through controlled immunostimulatory agent release [17,18]. The high water content, porosity, and elasticity of hydrogels optimize interactions with immune cells, promoting effective antigen presentation and uptake.

Hydrogels offer additional advantages over traditional delivery methods. Biocompatible polymers, such as polyvinyl alcohol (PVA), poly(ethylene glycol) (PEG), and chitosan, degrade safely after antigen release, minimizing the risk of toxicity or adverse immune reactions [15,19]. They also enable less invasive delivery methods, such as transdermal patches or microneedle arrays, making vaccines more accessible and acceptable. By combining targeted delivery with adjuvant properties, hydrogels hold the potential to redefine vaccine strategies for a range of diseases.

This review explores the potential of hydrogel-based vaccine delivery systems to enhance vaccine efficacy and immunogenicity, providing insights into hydrogel-guided delivery advances and their effectiveness in eliciting antigen-specific immune responses. We begin by discussing the role of vaccines in immunomodulation and hydrogels as adjuvants for targeted immune responses. We then contrast traditional delivery methods with innovative hydrogel-based approaches, including needle-free, microneedle, oral, and inhalable systems. Finally, we address challenges, including safety and scalability, highlighting opportunities to advance global immunization efforts.

## 2. Vaccine Adjuvants and Immunomodulation

Vaccination represents one of modern medicine’s most transformative achievements, harnessing the power of immunomodulation to protect against infectious diseases and enhance global health resilience [17]. By stimulating immune memory, vaccines have improved health outcomes for millions globally. Vaccines may be developed from inactivated or attenuated pathogens, surface molecules (e.g., carbohydrates, proteins, lipids), or recombinant antigens [18,20], all designed to elicit specific immune responses. Administered subcutaneously, intramuscularly, orally, or intranasally, these formulations engage the adaptive immune system, training it to recognize and neutralize pathogens. Traditional vaccines often consist of weakened or inactive pathogen components, which prompt antibody production and mobilize immune cells to eliminate the infectious agents [21]. This process strengthens immunological memory, enabling rapid and effective responses to future exposures to the same pathogen.

Despite the success of traditional vaccines, many exhibit weak immunogenicity, limiting their protective efficacy. To address this, formulations often incorporate adjuvants—substances that enhance and shape immune responses [22,23]. Effective adjuvants improve cost-effectiveness by reducing dose requirements, strengthening immune signaling, and promoting long-lasting immunity. Ideal adjuvants also support cytotoxic T-cell generation, high-specific antibody production, and antigen-specific clonal expansion, enhancing vaccine potency even at lower doses [24].

Advances in biotechnology have enabled the development of vaccines with highly purified recombinant antigens, thus prioritizing safety. However, such refined antigens often display reduced immunogenicity compared to live attenuated or inactivated pathogens. While some traditional vaccines contain inherent adjuvants due to their complex compositions [3], modern vaccine formulations often require deliberate incorporation of adjuvants to achieve targeted immune responses without compromising safety. This balance is particularly critical for vaccines administered to healthy populations. In therapeutic contexts, such as cancer vaccines, the risk–benefit analysis may favor more aggressive adjuvant strategies.

Currently licensed adjuvants in North America and Europe include aluminum salts, oil-in-water emulsions (e.g., MF59, AS03, and AF03), virosomes, and AS04, a combination of monophosphoryl lipid A and aluminum salt [25]. Adjuvants serve varied functions, from improving antigen delivery to stimulating innate immune receptors like Toll-like receptors (TLRs) [26]. Broadly, adjuvants are categorized into delivery systems, immunomodulatory molecules, and combination systems [3].

Immunomodulatory biologics, such as antigenic proteins, peptides, nucleic acids, and extracellular matrix components, are gaining recognition for their therapeutic potential across a range of immune-related conditions. These agents interact with immune cell receptors, such as TLRs on dendritic cells (DCs) or antigen-specific receptors on T and B cells, to guide the intensity, duration, and specificity of immune responses. By doing so, they offer a tailored approach to managing complex immune conditions [27,28].

Specific immunomodulators include ligands targeting innate immune receptors like TLRs, NOD-like receptors (NLRs), C-type lectins, and RIG-I-like receptors [29]. Examples include the TLR4 ligand MPL, used in the Cervarix HPV vaccine, and the TLR9 ligand CpG oligodeoxynucleotide (ODN), employed in Hepislav, a hepatitis B vaccine candidate [3,30]. The malaria vaccine RTS,S combines MPL and QS21 (a saponin-based adjuvant) in a liposomal formulation for enhanced presentation [31,32]. These combination systems, like AS01 in RTS,S and AS04 in Cervarix, demonstrate the synergy of multifaceted adjuvant approaches for enhancing efficacy.

Delivery systems, another major category of adjuvants, primarily improve antigen targeting and presentation. Liposomes, such as virosomes (liposomes with viral proteins) and niosomes (vesicles based on nonionic surfactants), carry antigens or immunomodulators either on their surface or encapsulated within, offering effective delivery without directly stimulating the immune system [32]. For example, the virosome-based vaccines Inflexal V (for influenza) and Epaxal (for hepatitis A) demonstrate the efficacy of such delivery systems [33].

Advanced formulations like squalene-based emulsions (e.g., MF59 and AS03) combine delivery and immunomodulatory properties, forming chemokine gradients that attract immune cells, monocytes and neutrophils, to the injection site and facilitate antigen uptake [34].

**Figure 1:**
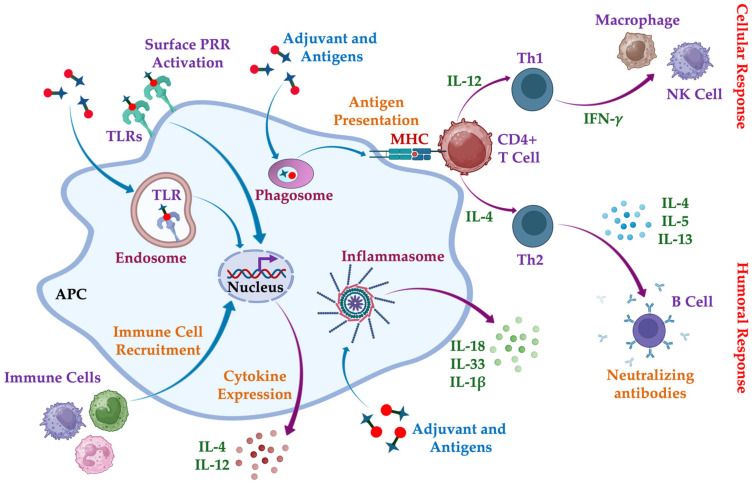
Adjuvants mediate their effects through mechanisms such as PRR activation, inflammasome-induced secretion of IL-1β and IL-18, and modulation of MHC antigen presentation. Some adjuvants may employ multiple pathways, including enhancing antigen uptake, PRR signaling, and immune cell recruitment. Created with BioRender.com, https://BioRender.com/m49n412 (accessed on 23 December 2024).

Adjuvants initiate immune responses through diverse mechanisms, often acting as ligands for pattern recognition receptors (PRRs) to initiate innate immunity [21,35]. This signaling triggers transcription factors that produce cytokines and chemokines, guiding responses like Th1 or Th2 and recruiting immune cells to the injection site, as illustrated in Figure 1 [3]. Some adjuvants activate the inflammasome, generating proinflammatory cytokines IL-1β and IL-18, while others enhance antigen presentation via MHC molecules [36]. Certain adjuvants, like alum, operate through multiple mechanisms, influencing antigen uptake, PRR signaling, inflammasome activation, and immune cell recruitment [3].

The versatility of adjuvants underscores their critical role in modern vaccines, especially those with highly purified antigens, enhancing immunogenicity and enabling tailored immune responses suited to both preventive and therapeutic applications.

## 3. Hydrogels as Vaccine Adjuvants

The development of effective vaccine adjuvants is critical to enhancing immunogenicity against diverse pathogens and in cancer immunotherapy [37,38,39]. An ideal adjuvant should simultaneously stimulate the immune system and facilitate antigen delivery [21]. While existing adjuvants enhance immune responses, challenges such as self-immunogenicity, safety, and biodegradability persist [40], underscoring the need for continuous research for safer and more efficient alternatives.

Hydrogels have emerged as promising candidates for vaccine delivery due to their unique ability to modulate antigen release kinetics while preserving their native conformation [41,42]. Compared to traditional bolus injections, hydrogels offer advantages such as high biomolecule-loading capacity, controlled release, and customizable degradation profiles. These properties make hydrogels particularly suitable for applications in cancer immunotherapy. However, practical challenges—such as the need for specific triggers (e.g., ultraviolet radiation, temperature changes, or enzymatic action) to initiate hydrogel formation—limit their utility in certain vaccination contexts and introduce safety concerns [43]. Additionally, the high cost and unproven safety of many immunostimulants hinder their broader adoption.

To address these challenges, research is increasingly focused on in situ injectable hydrogels that eliminate the need for external triggers alongside hydrogels with intrinsic adjuvant properties. Optimizing hydrogel systems for vaccine delivery requires addressing key considerations, such as the host immune response to the hydrogel biomaterials and the rational design of hydrogels to enhance immunomodulatory capabilities [44,45].

### 3.1. Host Immune Responses

The immune system, comprising innate and adaptive components, continuously monitors the body to detect tissue damage, pathogen invasion, or foreign materials. Humoral and cellular factors from both branches, including neutrophils, macrophages, dendritic cells (DCs), lymphocytes, and their secreted cytokines, coordinate to deliver an effective immune response [46]. These factors interact dynamically with hydrogels, initiating a foreign-body reaction (FBR) [47]. This reaction involves a cascade of inflammatory processes that result in fibrosis, cellular infiltration, and collagen deposition, which can influence hydrogel performance.

***Innate Immune Response*.** Upon implantation, proteins from blood and interstitial fluids adsorb onto the hydrogel surface within nanoseconds [48], with surface chemistry determining the type and extent of protein adsorption. For instance, negatively charged surfaces activate Factor XII, initiating the coagulation cascade and leading to thrombin release [49], platelets activation, and release of prothrombinases that assemble and activate on platelet surfaces, further amplifying thrombin formation and enhancing coagulation factor recruitment [50]. Concurrently, adsorbed fibrinogen, tissue factors, and complement proteins act as platelet adhesion substrates, fostering fibrin-rich clot formation. These clots serve as a transient matrix for cell adhesion and migration, providing cytokines and growth factors essential for reducing inflammation.

Neutrophils are the first responders [51], initiating phagocytosis and degranulation [44]. This leads to the release of proteolytic enzymes and reactive oxygen species (ROS), which are effective against pathogens but can degrade biomaterials. For example, hydrogels degrade under the enzymatic activity of neutrophil elastase. Neutrophils also release neutrophil extracellular traps (NETs), a network of granular proteins, elastase, chromatin DNA, and histones [52]. Excessive NET formation contributes to fibrotic matrix deposition. Additionally, neutrophils secrete cytokines such as interleukin-8 (IL-8), monocyte chemoattractant protein-1 (MCP-1), and macrophage inflammatory protein-1β (MIP-1β), recruiting monocytes, macrophages, DCs, and lymphocytes [53].

**Figure 2:**
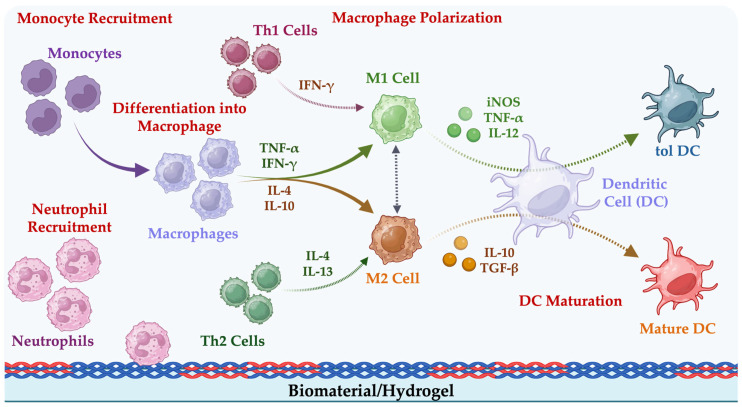
Illustration of immune cell dynamics upon hydrogel implantation, highlighting the recruitment of neutrophils during the first 48 h, followed by monocyte infiltration and differentiation into macrophages. Macrophage polarization into M1 (pro-inflammatory) or M2 (anti-inflammatory and reparative) phenotypes regulates inflammation and potential immunomodulation. Created with BioRender.com, https://BioRender.com/i53p251 (accessed on 23 December 2024).

Following neutrophil apoptosis, monocytes dominate the immune landscape, as shown in Figure 2. These monocytes secrete IL-1, IL-8, MCP-1, and MIP-1β, recruiting additional monocytes and driving their differentiation into macrophages. Macrophages play dual roles in inflammation and tissue regeneration by releasing inflammatory mediators, matrix-remodeling enzymes (e.g., metalloproteases), and fibroblast-stimulating growth factors. Depending on environmental cues, macrophages polarize into pro-inflammatory (M1) or anti-inflammatory (M2) phenotypes [54]. M1 macrophages, induced by interferon-γ (IFN-γ) and tumor necrosis factor-α (TNF-α), secrete cytokines such as nitric oxide synthase (iNOS), TNF-α, and IL-12, facilitating inflammation and adaptive immune recruitment. Conversely, M2 macrophages, stimulated by IL-4 and IL-10, promote wound healing, extracellular matrix (ECM) deposition, and angiogenesis. Persistent hydrogel presence may trigger macrophages to fuse into foreign-body giant cells (FBGCs), encapsulating the hydrogel into fibrotic tissue [55]. Macrophage polarization is dynamic and influenced by environmental signals, making them key targets for immunomodulation.

***Adaptive Immune Response*.** Dendritic cells (DCs), which bridge innate and adaptive immunity, play a critical role in hydrogel interactions. Synthetic hydrogels may induce tolerogenic DC phenotypes, promoting T-cell tolerance and reducing immune activation [56], whereas natural biomaterials like collagen, chitosan, or alginate promote DC maturation and T-cell activation [57]. Mature DCs stimulate T lymphocyte proliferation and cytokine secretion (e.g., TNF-α and IL-6), amplifying immune responses.

Close interactions between T lymphocytes and macrophages further modulate the immune response to foreign biomaterials. They regulate macrophage polarization, driving the switch from M1 to M2 phenotypes through shifts in cytokine profiles from Th1 to Th2 cells [58], and resolving inflammation. T lymphocytes and macrophages jointly release inflammatory mediators that recruit effector cells. Over time, these mediators are replaced by factors that support ECM remodeling, highlighting the importance of T lymphocyte–macrophage interactions in resolving inflammation during the FBR.

Hydrogel design must account for the activation of both innate and adaptive immune responses, as well as the intricate crosstalk between cellular and soluble immune components.

### 3.2. Rational Design of Hydrogels for Immunomodulation

Hydrogels physicochemical properties, including dimensionality, stiffness, porosity, topography, chemical composition, and surface characteristics [44,59], profoundly influence immune response. Rationally designing hydrogels to leverage these properties can optimize their immunomodulation and antigen-delivery functions.

***Dimensionality.*** Cells reside within a three-dimensional (3D) microenvironment formed by the extracellular matrix (ECM). Hydrogels that mimic this complex environment significantly impact immune cell behavior. For example, neutrophils in 3D hydrogels exhibit distinct morphologies, such as multiple pseudopods, compared to those on two-dimensional (2D) surfaces [60]. Similarly, monocytes exhibit spread shapes (~40–50 µm) on 2D plastic but adopt rounded forms (~20–30 µm) in 3D collagen matrices [61]. Dimensionality also affects immune functions, such as macrophage polarization and monocyte differentiation. THP-1 monocytes in 3D hydrogels spontaneously differentiate into CD68-positive macrophages, with enhanced nitric oxide (NO) and reactive oxygen species (ROS) production mediated by NF-κB pathway activation [62]. Moreover, the type of hydrogel influences polarization: PEG hydrogels favor pro-inflammatory M1 markers, whereas 3D lymph node ECM hydrogels promote anti-inflammatory M2 markers [63]. Such responses can be further modulated by additional stimuli, including cytokines like GM-CSF and lipopolysaccharides (LPSs). These findings underscore the importance of hydrogel dimensionality in modulating immune responses.

***Stiffness.*** Hydrogel stiffness, defined as its resistance to deformation, affects cellular signaling and immune modulation. While hydrogels are typically soft, their stiffness can be adjusted using cross-linkers or by altering polymer concentrations. Stiffer hydrogels (~12 kPa) promote neutrophil polarization and enhanced chemokine sensitivity, while softer hydrogels (~2 kPa) facilitate faster migration with random directions [64]. Stiffness also affects macrophage polarization: soft hydrogels favor the anti-inflammatory M2 phenotype characterized by interleukin-10 (IL-10) secretion, whereas stiff hydrogels promote the pro-inflammatory M1 phenotype with higher expression of inflammatory cytokines [65,66,67]. Similarly, soft hydrogels induce weaker FBR in vivo, forming thin fibrous capsules compared to the dense layers formed around stiff hydrogels. Adaptive immune cells are mechanosensitive as well; T lymphocytes on stiffer hydrogels exhibit enhanced spreading, migration, and glycolytic activity driven by CD3 signaling [68]. B lymphocytes also show stronger activation on stiffer hydrogels, attributed to microtubule-dependent mechanosensing [69]. These mechanotransduction properties highlight the significance of tailoring hydrogel stiffness to match native tissue properties for effective immunomodulation.

***Porosity.*** The pore size and structure of hydrogels regulate nutrient exchange, waste diffusion, and immune cell infiltration. Larger pores facilitate deeper immune cell migration, enabling effective modulation of immune responses [70]. For example, hydrogels with 50% degradable porogen exhibited greater dendritic cell (DC) infiltration compared to those with 25% or 75%, with infiltrated DCs maintaining an immature phenotype suitable for further programming [71]. Conversely, nanoporous hydrogels can prevent immune cell infiltration, shielding encapsulated biomolecules. Porosity also influences macrophage polarization. Hydrogels with larger pores tend to favor M1-to-M2 transitions, supporting anti-inflammatory cytokine production and angiogenesis [72]. However, the relationship between porosity and immune response remains complex and context-dependent, necessitating further research for optimal design.

***Surface Topography.*** Micropatterned and nanopatterned hydrogels, created through techniques such as soft photolithography, influence macrophage alignment and phenotype [73]. For instance, microgrooved hydrogels enhance the secretion of anti-inflammatory markers, such as IL-1RA, and improve phagocytic activity compared to micropillar or unpatterned hydrogels [74]. Nanopatterned surfaces, fabricated using silicon molds, reduce fibrotic capsule formation and macrophage infiltration, likely by minimizing cell adhesion [75]. These findings demonstrate the potential of surface topography to fine-tune immune responses for targeted therapeutic applications.

***Chemical Composition and Surface Properties.*** The chemical properties of hydrogels, including hydrophobicity, wettability, charge, and functional group presentation, significantly impact immune responses. For example, hydrophobic surfaces have been shown to promote M1-like phenotype with enhanced cytokine secretion, while hydrophilic surfaces favor M2-associated cytokine production and macrophage behavior [76]. Similarly, positively charged hydrogels enhance macrophage spreading and fusion, whereas negatively charged hydrogels reduce foreign body responses [77]. These properties can be tailored to achieve desired immune outcomes.

The rational design of hydrogels is critical to ensure effective immunomodulation [44,78]. By tailoring their unique properties, hydrogels can be engineered to present biochemical and biophysical cues and modulate immune responses with precision. Their integration into vaccine delivery systems offers opportunities to enhance antigen efficacy, minimize adverse reactions, and address challenges in immune modulation.

## 4. Conventional Vaccine Administration Methods and Their Limitations

The effectiveness of vaccination hinges on delivery methods that elicit robust immune responses and establish long-lasting immunological memory. Conventional vaccine administration methods—both parenteral (e.g., intramuscular, subcutaneous) and non-parenteral (e.g., oral, intranasal)—play a critical role in shaping vaccine outcomes [16]. Factors such as proximity to lymph nodes, antigenic epitope presentation, and adjuvant functionality are key to optimizing immune responses [21] and reducing the risk of vaccine failure.

### 4.1. Parenteral Delivery Methods

Parenteral methods, including intradermal (ID), subcutaneous (SC), and intramuscular (IM) delivery, remain the most common approaches for vaccine administration. The choice of administration site depends on anatomical factors, patient age, and vaccine type [79]. For adults, the deltoid region is typically preferred for intramuscular or intradermal injections, while the outer triceps area is common for subcutaneous delivery [80,81,82]. In contrast, the anterolateral thigh region is preferred for infants and toddlers [83]. Needle selection is tailored to tissue thickness, muscle size, and body mass to ensure accurate and effective antigen delivery.

While these methods are effective, they have notable limitations. The use of needles poses challenges such as pain, needle-stick injuries, the need for skilled personnel, and risks of disease transmission due to contaminated needles [84]. Another drawback is that vaccines administered via SC or IM routes are deposited into subcutaneous fat or muscle, which contain relatively few dendritic cells (DCs). These immune cells are essential for antigen uptake, processing, and presentation to T lymphocytes in lymphoid organs [85]. In contrast, the dermis and epidermis are densely populated with DC subsets, making the skin a highly effective site for vaccination [86]. Skin-based delivery can elicit stronger immune responses with lower levels of antigen, offering greater efficiency and saving resources [87].

### 4.2. Non-Parenteral and Needle-Free Methods

Alternative delivery methods, such as oral, intranasal, and transcutaneous routes, show promise for enhancing vaccine efficacy [88]. These routes can stimulate localized immunity and enhance protection directly at primary infection sites, such as the respiratory or gastrointestinal mucosa [89]. For example, oral vaccines target gastrointestinal immunity, while intranasal vaccines focus on respiratory defenses [90]. Transcutaneous methods use patches or microneedles to deliver antigens through the skin, offering advantages like ease of use, reduced cold chain dependence, and minimized patient discomfort.

Needle-free systems, including jet injectors and powder or liquid propulsion devices, eliminate needle-stick injuries, reduce pain, and minimize the need for skilled personnel [91]. These approaches are particularly advantageous for large-scale immunization campaigns, especially in resource-limited settings where logistics and cold chain requirements often pose barriers.

### 4.3. Addressing Limitations Through Innovation

Despite their utility, conventional methods have challenges, such as pain, risk of disease transmission from contaminated needles, and logistical hurdles in mass vaccination efforts. To overcome these issues, researchers have developed innovative delivery systems that enhance immunogenicity, stability, and patient acceptance.

- **Nucleic acid-based systems**: for example, DNA vaccines, which introduce genetic material to produce antigens in vivo, eliciting robust immune responses [92].
- **Adjuvant-enhanced delivery**: adjuvants amplify immune activation, improving vaccine efficacy.
- **Microparticle-based systems**: antigens are encapsulated to protect them from degradation and enable controlled release, providing prolonged immune stimulation [21,93].

The effectiveness of these strategies depends on their ability to elicit appropriate immune responses and establish immunological memory. The closer the vaccine is delivered to lymph nodes or lymphatic vessels, the stronger the immune response. Factors like antigenic epitopes and adjuvant functionality also play crucial roles in determining the outcome of vaccination. The delivery site significantly influences vaccine efficacy. Understanding these nuances enables the design of tailored delivery systems to maximize vaccine effectiveness.

### 4.4. Future Directions

Efficient vaccine delivery systems are crucial for the success of immunization programs. While traditional parenteral methods remain foundational, their limitations underscore the need for innovative non-parenteral and needle-free solutions. By integrating advanced technologies like microparticles, nucleic acids, and adjuvants, researchers aim to revolutionize vaccine delivery. These advances promise to enhance patient acceptance, minimize risks, expand global vaccine accessibility, and improve protection against infectious diseases.

## 5. Polymeric Hydrogels for Vaccine Delivery

Polymeric hydrogels are emerging as innovative vaccine delivery platforms, offering controlled antigen release and enhanced immune responses that address the limitations of conventional vaccination methods. These hydrogels, derived from both natural and synthetic polymers, form supportive matrices capable of encapsulating antigens or adjuvants. This encapsulation facilitates the targeted delivery and prolonged antigen exposure, which enhances immunogenicity and promotes long-term immunity.

Natural polymers, such as chitosan, alginate, and agarose, are favored for their biocompatibility and biodegradability, making them suitable for mucosal and oral vaccine delivery. Synthetic polymers, including poly(N-isopropylacrylamide) (PNIPAAm), polycaprolactone (PCL), poly(ethylene glycol) (PEG), and poly(lactic-co-glycolic acid) (PLGA), provide precise control over degradation rates and sustained antigen release, improving immune responses. A comparison of polymer types, administration routes, and targeted diseases is summarized in Table 1.

Unlike traditional vaccine delivery methods, which typically only cause short-term antigen exposure and often require multiple doses to trigger long-term immunity, polymeric hydrogels support extended antigen exposure leading to increased germinal center (GC) activity, where B cells undergo selection and affinity maturation. The longer GC response leads to higher-quality, high-affinity antibodies, and better immune memory. For instance, a self-healing polymer–nanoparticle (PNP) hydrogel vaccine was shown to deliver antigens to mice over several, significantly enhancing the strength, persistence, and efficacy of immunogenicity compared to conventional bolus injection [41]. Similarly, a hydrogel-based nanoparticle scaffold for a SARS-CoV-2 subunit vaccine promoted potent GC reactions, resulting in robust antibody generation and T-cell responses [94].

**Table 1:** Overview of natural and synthetic polymers, their administration routes, and targeted diseases/viruses/bacteria.

Polymer	Route	Antigen	Reference
**Natural**			
Chitosan	Nasal	*Streptococcus pneumoniae*	[95]
Chitosan		Plasmid DNA-HPV E7	[96]
Hyaluronic acid	Injectable	SARS-CoV-2	[94]
Dextran	Nasal	Tetanus-mixed toxoid	[97]
Dextran	Injectable	Murabutide and Ovalbumin	[98]
Hyaluronic acid	Nasal	Ovalbumin	[99]
Chitosan	Nasal	SARS-CoV-2	[100]
Alginate	Intradermal	*Bacillus Calmette*–Guérin (BCG)	[101]
**Synthetic**			
PLA		Recombinant Fusion protein (TV) and Filarial epitope protein (FEP)	[102]
PLA	Intramuscular	Hepatitis B Surface Antigen	[103]
PEG	Intramuscular	Ovalbumin	[104]
PCL	Intranasal	Ovalbumin	[105]
PCL		*Leishmania infantum*	[106]

### 5.1. Natural Polymers

Natural polymers are increasingly being investigated for vaccine delivery due to their inherent biocompatibility, biodegradability, and unique immunomodulatory properties. These polymers act as both delivery vehicles and adjuvants, enhancing immune responses while minimizing the need for synthetic additives.

***Chitosan (CS)***, a derivative of chitin, is extensively studied for its ability to form electrostatic complexes with negatively charged antigens due to its natural positive charge [106,107,108,109]. This interaction promotes cellular uptake and stimulates both humoral (antibody-mediated) and cellular (T-cell mediated) immunity, making it particularly effective against pathogens like Streptococcus pneumoniae [95]. For example, CS nanocapsules designed with a thiol–maleimide linkage for presenting the PsaA antigen, common across pneumococcus serotypes, demonstrated a threefold increase in antigen uptake and a robust immune response, including T-cell activation and cytokine (TNFα) secretion [96]. Similarly, (CS)-tripolyphosphate (TPP)-plasmid DNA (pDNA) nanoparticles have shown promise in nucleic acid delivery, enhancing the expression of antigens like E7 in human cells for SARS-CoV-2 and HPV vaccines [110].

***Alginate***, a hydrophilic polymer known for its high biocompatibility and mucoadhesive properties, supports controlled antigen release and increased immune cell uptake [111,112,113,114]. The higher water solubility and poor mechanical properties of alginate, however, limit its effectiveness as an antigen carrier. Crosslinking of alginate with other natural or synthetic polymers is needed to overcome these limitations [115,116]. Injectable chitosan-alginate gel scaffold loaded with mRNA lipoplexes significantly elevated T cell proliferation and IFN-γ secretion, resulting in a fivefold higher immune response compared to systemic injection [114]. This scaffold also provided protection from stomach acid degradation, ensuring antigen stability and sustained release, which led to enhanced mucosal immunity and prolonged immune response [113].

***Dextran***, derived from bacterial sources, is hydrophilic and forms bioadhesive hydrogels ideal for vaccine delivery. Cross-linked dextran microspheres can overcome mucosal barriers, decreasing the mucociliary transport rate and increasing residence time in the nasal cavity, leading to a stronger immune response. The ability to formulate cross-linked dextran as dry powder results in prolonged shelf time [96]. Acetalated dextran (Ace-Dex) has demonstrated tunable acid-sensitive properties, facilitating controlled antigen release and heightened immunogenicity. For example, Ace-Dex microparticles loaded with murabutide and ovalbumin (OVA) enhanced antibody and cytokine production, offering a flexible and effective vaccine delivery system [117].

### 5.2. Synthetic Polymers

Synthetic polymer hydrogels have great potential in vaccine delivery due to their tunable physical and chemical properties, controlled degradation rates, and ability to enhance immune responses.

***Poly(lactic acid)*** (PLA) is a biocompatible polymer commonly used in several medical applications. PLA microparticles have successfully been used to carry antigens in single-dose vaccines for multiple diseases, including hepatitis B and lymphatic filariasis. In a recent study, Fusion protein (TV) and Filarial epitope protein (FEP) encapsulated in PLA triggered significant antibody production in mice with a single dose [102]. Although PLA is biodegradable in vivo, its rigid main chain and the presence of methyl groups on its side chains result in a slow degradation rate.

***Poly(lactic-co-glycolic acid)*** (PLGA) combines the advantages of PLA and poly(glycolic acid) (PGA), offering slow antigen release and a high safety profile. While PGA degrades into glycolide, causing a strong inflammatory response, its co-polymerization with PLA improves its safety profile and provides high adjuvant properties. For example, PLGA-encapsulated vaccines for H9N2 Influenza A virus induced strong Th1 and Th2-type cytokine responses, alongside significant CD4+ and CD8+ T cell activation, providing a robust and sustained cellular and humoral immune response in chickens [118]. In this study, 15% of the antigens were released during the first 12 h after administration, while the remaining 85% were slowly released over 40 days. Histological analysis confirmed no toxicity or inflammation with a robust and long-lasting immune response.

***Poly(ethylene glycol)*** (PEG) is a biocompatible polymer extensively studied for vaccine delivery, including mRNA-based COVID-19 vaccines. However, its use is limited by weak immunogenicity and the potential for anti-PEG antibodies causing allergic reactions [119,120,121,122,123]. Current research aims to address these challenges by modifying PEG formulations to improve immunogenicity and safety. Reducing the density and size of PEG nanoparticles prevents their recognition by the immune cells and IgM antibodies [124]. In addition, cross-linking with hyaluronic acid or chitosan can reduce the adverse immune responses to PEG [125,126].

***Polycaprolactone*** (PCL) offers excellent biocompatibility and very slow degradation, making it suitable for long-term antigen release. These properties reduce the frequency of vaccine administration, facilitate vaccination logistics, and ameliorate the side effects associated with rapid antigen release [127,128]. PCL nanoparticles loaded with tetanus toxoid antigen triggered sustained immune memory responses involving CD4+ and CD8+ T cells, even with just a single dose administration [129].

***Poly(N-isopropylacrylamide)*** (PNIPAAm) is a thermosresponsive polymer capable of reversible phase transitions, enabling unique pathways for antigen delivery. For instance, PNIPAAm nanogels were used to encapsulate a recombinant virulence protein from Actinobaccilus pleuripneumoniae for oral vaccination, successfully delivering antigens to the lungs and stimulating robust immune responses [130]. This was accomplished by leveraging the ability of PNIPAAm to change from liquid to sol phase at about 32–37 °C. Crosslinked hydrogels of PNIPAAm and chitosan formed a stable network at body temperature, allowing efficient and prolonged vaccine release [131].

Overall, the unique advantages and disadvantages of natural and synthetic polymers play a critical role in determining their suitability for vaccine delivery platforms. Natural hydrogels offer superior biocompatibility, biodegradability, and bioactivity, closely mimicking the extracellular matrix to support cellular interactions. However, they often suffer from poor mechanical strength, inconsistent batch-to-batch properties, and rapid degradation under physiological conditions. Synthetic hydrogels, on the other hand, provide tunable mechanical properties, consistent composition, and controlled degradation rates, making them ideal for precise vaccine delivery [132,133]. Despite these strengths, their lack of inherent bioactivity, potential inflammatory responses, and toxicity from degradation by-products pose challenges. A comparison of specific advantages and disadvantages of natural and synthetic hydrogels for vaccine delivery platforms is summarized in Table 2.

## 6. Hydrogel-Guided Vaccine Delivery Systems

Hydrogel-guided vaccine delivery systems represent a transformative advancement in immunization, offering viable alternatives to traditional injection-based methods. These novel systems leverage the unique properties of hydrogels—such as high water content, biocompatibility, and tunable mechanical and chemical characteristics—to enable effective transdermal, intranasal, mucosal, and oral administration. Such approaches not only improve patient acceptance but also expand vaccination accessibility, particularly in resource-limited settings where maintaining cold chain logistics is challenging. By combining controlled antigen release with targeted immune activation, hydrogel-guided delivery systems pave the way for effective, painless, and patient-friendly immunization strategies.

### 6.1. Needle-Free Mucosal Vaccine Delivery

Needle-free mucosal vaccine delivery systems aim to administer vaccines through the mucosal surfaces without requiring needles, offering benefits such as reduced medical waste and ease of administration. These systems operate in two stages: (1) the mucoadhesive polymer adheres to the mucus membrane and (2) vaccine release is triggered by polymer degradation, facilitated by moisture at the administration site. Mucosal surfaces, supported by vascular and lymphatic drainage, promote antigen absorption and trigger both cellular and humoral immune responses [134].

***Oral route***. Oral vaccine delivery targets the gastrointestinal tract (GIT) to generate immune responses, overcoming challenges posed by the harsh GI environment, including acidic pH and enzymatic degradation. Hydrogels composed of natural polymers, such as chitosan, dextran, and alginate, as well as synthetic polymers like dimethylaminoethyl methacrylate (DMAEMA), have been developed for this purpose [135].

For instance, alginate-coated layered double hydroxide nanoparticles were shown to protect antigens in the stomach and enable controlled release in the intestines, enhancing uptake by gut-associated lymphoid tissue (GALT) and macrophages, which improved immune responses [111]. Similarly, chitosan-based hydrogels demonstrated strong mucosal adhesion and prolonged antigen retention in the intestinal lining, boosting immune activation. These hydrogels also showed high antigen loading efficiency and stability, leading to significant macrophage uptake and enhanced immune responses [136].

**Figure 3:**
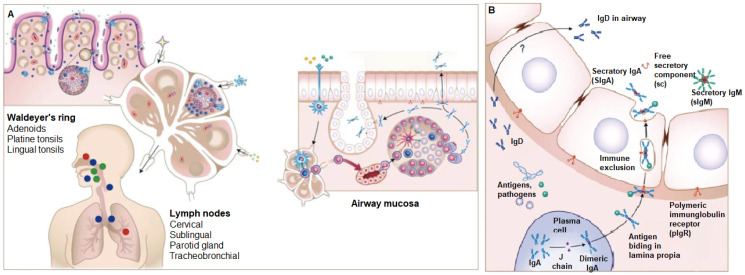
Overview of respiratory mucosal immune components: Waldeyer’s ring (green), lymph nodes (blue) for systemic IgG responses, and ectopic tissues (red) like NALT and BALT for IgA production. (**A**) Dimeric IgA production in mucosal sites (BALT and NALT) facilitates immune exclusion by expelling antigens and involving pIgR-dependent secretory IgM and IgD (**B**). Reproduced with permission from Heida et al., 2022 [142].

***Intranasal route***. Intranasal delivery systems exploit the respiratory tract’s mucosa-associated lymphoid tissues (MALTs) to induce local IgA-mediated immune responses. These responses mimic natural infection patterns, promoting faster virus neutralization upon reinfection and potentially reducing the need for a booster (Figure 3).

A chitosan-based hydrogel matrix to deliver antigens to the upper respiratory tract showed increased nasal mucosa tissue-resident memory and an enhanced immune response [137]. Similarly, an inhaled subunit vaccine candidate targeting the SARS-CoV-2 Spike S1 protein provided enhanced protection against severe infection when administered intranasally and intratracheally [138]. Dry powder formulations of inhalable vaccines further simplify logistics by eliminating cold chain requirements, making them promising solutions for pathogens like Mycobacterium tuberculosis, Human papillomavirus, influenza A, and SARS-CoV-2 [100,135,138,139,140,141].

### 6.2. Hydrogel Patch-Based Delivery

Hydrogel patch-based systems deliver vaccines transcutaneously, enabling antigens to penetrate the skin’s immune cell-rich layers. The skin hosts various immunocompetent cells, including Langerhans cells (LCs), keratinocytes, mast, and dermal dendritic cells. LCs in particular are strong antigen-presenting cells (APCs), playing a key role in defending against foreign antigens. Thus, it is expected that the direct exposure of LCs to antigens will produce an effective immune response. However, because the stratum corneum acts as an effective physical barrier, antigenic proteins applied to naked skin are unable to reach LCs in the epidermal layer. Hydrogel patches facilitate antigen penetration through the stratum corneum (SC) [143]. One study demonstrated that antigens delivered via a hydrogel patch were captured by LCs and transported to local lymph nodes, effectively activating the immune system [144].

Patch-based systems also address challenges in generating immune memory CD8+ cytotoxic T-lymphocytes (CTLs), which are essential for combating infections and malignancies. For instance, a hydrogel patch co-delivering ovalbumin and mXCL1-V21C/A59C significantly enhanced immune memory of CD103+ DCs at the application site and adjacent lymph nodes, improving antigen presentation and significantly increasing memory CTL responses [145].

### 6.3. Microneedle-Based Delivery

Microneedle (MN) patch-based systems employ minimally microneedle arrays for transcutaneous vaccine delivery, offering a safer and more patient-friendly alternative to traditional syringes. MN patches penetrate the skin barrier effectively, targeting APCs like dendritic cells and macrophages in the epidermis and dermis, resulting in robust immune responses. Polymers such as hyaluronic acid (HA), polyvinylpyrrolidone (PVP), and chitosan are commonly used for MN fabrication [146].

**Figure 4:**
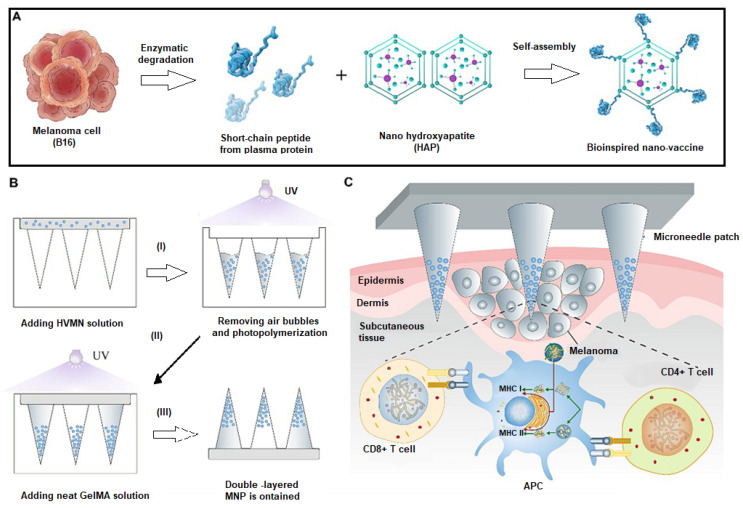
Preparation and application of double-layered MNP: (**A**) nano-vaccine with HAP and melanoma peptides, (**B**) mold-cast double-layered MNP, and (**C**) dual-function MNP for anti-tumor and regenerative melanoma treatment. Reproduced with permission from Chen et al. (2024) [149].

***Applications in Cancer Vaccines***. MN-based nanovaccines are gaining prominence for cancer immunotherapy, enhancing anti-tumor immunity and preventing tumor recurrence and metastasis. These nanovaccines promote lymph node drainage, APC assimilation and maturation, and T cell recognition [147,148]. For instance, a double-layer MN patch using methacrylated gelatin (GelMA)-based was effectively used to develop a therapeutic vaccine against malignant melanoma [149]. The MN tips were loaded with short-chain peptides from tumor cells conjugated into self-assembling hydroxyapatite (HAP) nanoparticles as illustrated in Figure 4. Significant biocompatibility and immune activity were demonstrated, effectively suppressing malignant melanoma growth, and triggering apoptosis, while supporting tissue regeneration [149].

***Thermally Stabilized Vaccines***. Thermally stable vaccines that do not require cold-chain storage can simplify logistics and speed up response, especially during pandemics and natural disasters. For instance, a recombinant SARS-CoV-2 S1-RBD protein stabilized in trehalose-sucrose (TS) was encapsulated in a shell made of PLGA and loaded into a single-administration, programable pulsatile-release MN patch capable of releasing the antigen on the implantation site over an extended period. Extended antigen release and protein stability was demonstrated in a murine model for at least 4 months, leading to an immunogenic effect equivalent to that induced by multiple bolus injections [150].

### 6.4. Influence of Vaccine Type on Hydrogel Selection

The choice of a hydrogel delivery system depends on the type or generation of vaccine, as each has unique requirements for stability, release kinetics, and immune activation. With a wide range of materials and delivery systems, hydrogels can provide tailored solutions for preserving vaccine efficacy and enhancing delivery for first-, second-, and third-generation vaccines.

***First-Generation Vaccines*** (Live Attenuated or Inactivated Pathogens) rely on the structural and functional integrity of their biological components, such as live attenuated or inactivated viruses, to elicit an immune response [151]. Hydrogels play a crucial role in maintaining the viability of live organisms or the integrity of inactivated pathogens during storage and delivery. Encapsulation in hydrogel matrices sustains antigen release, prolonging exposure to the immune system and boosting immunogenicity [12]. The crosslinked density of the hydrogels affects permeability and release rate, making it a good target for optimization. For example, live Zika virus entrapped in chitosan-based hydrogel cross-linked with nano-CaCO_3_ and polyaspartic acid induced robust antigen-specific adaptive responses and immune memory. The strong interaction between the Zika virus and the hydrogel ensured that no live virus was released from the scaffold [152].

***Second-Generation Vaccines*** (Subunit or Recombinant), which use specific protein antigens or recombinant components [41], require protection against degradation and environmental stresses. Hydrogels enable the encapsulation and stabilization of these antigens, preserving their bioactivity. Moreover, the controlled and sustained release from hydrogel-based systems optimizes presentation to APCs and enhances immune activation. The structure and crosslinking of hydrogels can be tuned to optimize antigen release kinetics. For instance, the effect of antigen release kinetics on the immune response to a subunit-based vaccine encapsulated in a PEG-based hydrogel was investigated by varying the linkage type [153]. Hydrogels with thioether linkages provided a sustained release of antigens, resulting in enhanced antigen presentation and immune response, compared to hydrogels with disulfide linkages, which provided a more rapid release of antigens.

***Third-Generation Vaccines*** (DNA or mRNA), present unique challenges for delivery, such as protection against enzymatic degradation while facilitating cellular uptake [154]. Hydrogels address these challenges by providing a protective matrix for nucleic acids, maintaining their biological activity, and facilitating their sustained release and precise delivery, improving immunogenicity. Adjusting the hydrogel particle size, chemistry, hydrophilicity, crosslinking density, and pore size can help to optimize nucleic acid protection and the release profile [155].

## 7. Vaccine Safety and Regulatory Challenges

The advent of nanotechnology has significantly influenced hydrogel-guided vaccine delivery systems, offering innovative solutions to enhance immune responses. Despite extensive research on nanotechnology-driven platforms, the commercial availability of nano-based adjuvants remains limited partly due to safety concerns and regulatory constraints.

### 7.1. Challenges in Commercialization and Clinical Development

While nanotechnology offers tremendous potential, significant challenges hinder the commercialization and clinical translation of such vaccine delivery systems. Critical gaps exist in understanding the effects of physicochemical properties and mechanisms of action on bioavailability, intracellular signaling, cellular uptake, and internalization [156]. These uncertainties necessitate comprehensive investigations to ensure the reliability and safety of hydrogel-guided vaccine delivery platforms. Additionally, rigorous toxicology studies must be conducted on vaccine batches produced with final manufacturing methods and raw materials. Manufacturers are required to provide preclinical data on local tolerance and repeated-dose toxicology in both male and female animal models. Parameters such as animal weight, feed intake, hematological status, local reactions, and immunological changes must be evaluated [12]. The World Health Organization (WHO) further mandates histological examinations of tissues, particularly at the injection site, for vaccines incorporating novel adjuvants [157].

### 7.2. Addressing Hydrogel and Biomaterials Toxicity and Safety Concerns

The unknown safety profile of hydrogels and other novel biomaterials presents a double-edged sword in biomedical applications. While these materials offer advantages due to their biocompatibility and capacity for controlled delivery, they may induce adverse effects depending on the administration route. For instance, dermal administration of hydrogel-based systems may lead to dermatological issues, oral delivery could cause gastrointestinal disturbances, and nasal or parenteral routes may provoke respiratory or cardiovascular complications [134,157,158]. Moreover, hydrogels capable of crossing biological barriers, such as the blood–brain barrier, could potentially affect neural tissues.

Studies on animal models indicate that prolonged exposure to biomaterials may lead to cellular accumulation or inflammatory responses, raising concerns about long-term toxicity [159]. Specific components used in biomaterials, such as saponin-based adjuvants in immunostimulant complexes (ISCOMs), remain under scrutiny due to unresolved safety issues [160]. These challenges highlight the importance of continued research and caution when incorporating hydrogels and other biomaterials into vaccine formulations.

### 7.3. Regulatory Progress and Limitations

Although a growing number of nanomedicines and biomaterial-based systems have reached clinical trials, only a few have received FDA approval [161,162]. These include chemotherapeutics, antimicrobial agents, and treatments for autoimmune, psychological, and other medical conditions. However, the development and commercialization of these technologies are constrained by several factors, including the lack of clear definitions, comprehensive characterization protocols, and effective regulatory frameworks.

The interactions of hydrogels and biomaterials with biological systems depend on their structural and functional properties, including composition, surface coating, and potential aggregation. Aggregated biomaterials may exhibit properties distinct from individual particles, resulting in unpredictable toxic effects. The absence of standardized protocols for evaluating these properties limits early identification of toxic potential, often leading to late-phase clinical trial failures.

### 7.4. Strategies for Improving Regulatory Approaches

To overcome these challenges, regulatory agencies have adopted frameworks like those used for conventional drugs. Nanomedicines and biomaterial-based systems must attain Investigational New Drug (IND) status to initiate clinical trials, which proceed in three phases [163]. Phase 1 evaluates safety, phase 2 assesses efficacy, and phase 3 examines safety, efficacy, and dosage. Successful completion of these phases allows for FDA approval [163,164]. However, this conventional regulatory approach has faced criticism for failing to address the unique complexities of nanotechnology and biomaterials. Given the rapid advancements in nanotechnology and biomaterial sciences, a reformed regulatory framework is urgently needed. This framework should involve international collaboration to establish specific, rigorous guidelines addressing the safety and efficacy of these materials. Such protocols must consider the unique physicochemical properties of hydrogels and biomaterials and their potential interactions with biological systems.

### 7.5. Toward Safer and More Effective Vaccine Delivery Systems

Nanotechnology and biomaterials have seen dynamic growth, with increasing investments in research and development worldwide. Despite the uncertainties surrounding the practical application of these technologies, they hold immense promise. Researchers must prioritize comprehensive safety assessments, including toxicology, compatibility studies, and detailed evaluations of material interactions with biological systems. Improved regulatory cooperation among agencies can simplify the approval process, reducing delays in bringing nano-based and biomaterial-based medicines to market. As governments and stakeholders strive to refine regulatory frameworks, the potential of hydrogels and biomaterials to revolutionize vaccine delivery becomes increasingly apparent. Addressing safety concerns through robust research and regulation will enable the safe and effective integration of these advanced materials into vaccine delivery, ultimately benefiting public health on a global scale.

## 8. Challenges and Opportunities in Hydrogel-Guided Vaccine Delivery Systems

A successful vaccine delivery mechanism is critical for eliciting a robust immunological response, ensuring maximum vaccine efficacy, and inducing significant antigen-mediated immune reactions. Hydrogel-guided vaccine delivery systems hold immense potential to revolutionize immunization through precise, targeted, and patient-friendly antigen administration. However, several key barriers must be addressed to facilitate their widespread application [16]. The primary challenges include the following:

(i) **Enhanced immune response**: the delivery system must significantly boost antigen-mediated immune responses, ensuring strong and sustained immunogenicity.
(ii) **Biocompatibility and safety**: hydrogels must exhibit high biocompatibility, avoiding inflammatory or adverse immunogenic reactions while preserving structural integrity throughout degradation.
(iii) **Effective delivery**: Achieving controlled and efficient antigen delivery to elicit both humoral and cell-mediated immunity is vital. Long-term protection requires a balance of high-titer neutralizing antibodies from plasma cells and the activation of memory B and T cells.

One major technical challenge lies in achieving optimal antigen release kinetics. A release profile that mimics conventional bolus injections (too rapid) may diminish the delivery system’s benefits, while excessively delayed release could compromise immune activation. Innovative designs leveraging responsive materials—such as hydrogels sensitive to environmental factors like pH, temperature, or enzymatic activity—are essential for tailoring release dynamics.

Scalability and manufacturing costs are also critical barriers. Advanced hydrogels often require specialty polymers or intricate designs, leading to high manufacturing costs and challenges in large-scale production. These factors can limit the feasibility of widespread deployment, particularly in resource-constrained settings.

Despite these challenges, hydrogel-guided vaccine delivery systems present numerous opportunities. Their ability to sustain antigen release reduces the need for booster doses, improving patient acceptance and easing logistical constraints. Moreover, needle-free administration methods, such as transdermal or mucosal routes, minimize pain, mitigate needle-associated risks, and enhance accessibility –especially for pediatric and needle-phobic populations [134]. Additional advantages include the following:

(i) **Biodegradability**: hydrogel polymers degrade naturally within physiological environments, minimizing risks of tissue cytotoxicity.
(ii) **Immunogenicity**: carefully designed hydrogel systems can significantly enhance immune responses compared to free antigens.
(iii) **Smart material innovations**: recent advances in hydrogel materials, such as self-healing and stimuli-responsive properties, are paving the way for adaptable platforms tailored to specific immunization needs [165].

While promising immune responses and efficiency have been demonstrated in certain hydrogel-based vaccines, further research is required to enhance mechanical stability and achieve consistent long-term immunogenicity. Addressing these limitations will require multidisciplinary collaboration across immunology, materials science, and biomedical engineering. Future efforts should focus on the following:

(i) Developing cost-effective manufacturing techniques to support large-scale production.
(ii) Customizing hydrogel properties for targeted delivery tailored to specific diseases.
(iii) Advancing hydrogel-based cancer vaccines, including innovative preparation techniques and strategies to ensure affordability and scalability.

By addressing these challenges, hydrogel-guided vaccine delivery systems have the potential to transform public health outcomes, making vaccinations safer, more effective, and universally accessible.

## 9. Conclusions

Hydrogel-based vaccine delivery systems represent an innovative advancement in immunization, providing enhanced antigen targeting, controlled release, and improved immunogenicity. Leveraging their biocompatibility, tunable properties, and capacity to elicit robust immune responses, hydrogels address many limitations of traditional vaccine delivery methods. These systems not only improve vaccine efficacy but also support needle-free, patient-friendly administration routes, such as mucosal and transdermal delivery, broadening accessibility and reducing patient discomfort. Despite challenges such as scalability, production costs, and regulatory complexities, ongoing multidisciplinary research is continually refining hydrogel formulations and their applications. The incorporation of hydrogels into vaccine development has the potential to revolutionize immunization strategies, making vaccines safer, more effective, and more accessible across diverse populations and for a wide variety of diseases.

## Figures and Tables

**Table 2:** Comparison of natural and synthetic polymers for vaccine delivery platforms, highlighting their advantages and disadvantages.

Type	Advantages	Disadvantages
Natural Polymers	‑ High biocompatibility and biodegradability ‑ Possess cell-interactive groups enhancing attachment and proliferation ‑ Low immunogenicity and toxicity ‑ Bioactive properties aid vaccine effectiveness ‑ Mimic extracellular matrix (ECM) structure ‑ Degradable by natural enzymes	‑ Poor mechanical properties ‑ Inconsistent properties due to batch-to-batch variations ‑ Risk of rapid degradation under physiological conditions ‑ Susceptibility to pathogen contamination ‑ Limited control over properties
Synthetic Polymers	‑ High tunability for mechanical and chemical properties ‑ Consistent composition ‑ Long shelf life ‑ Adjustable degradation rates for controlled vaccine release ‑ Potential for customized drug delivery	‑ Lack of inherent bioactivity requiring modifications ‑ Potential for inflammatory responses ‑ Toxic degradation by-products in some cases ‑ Less compatibility with biological systems compared to natural polymers

## Data Availability

No new data were created or analyzed in this study.
